# Understanding the Links among *neuromedin U* Gene, *beta2-adrenoceptor* Gene and Bone Health: An Observational Study in European Children

**DOI:** 10.1371/journal.pone.0070632

**Published:** 2013-08-01

**Authors:** Francesco Gianfagna, Daniela Cugino, Wolfgang Ahrens, Mark E. S. Bailey, Karin Bammann, Diana Herrmann, Anna C. Koni, Yiannis Kourides, Staffan Marild, Dénes Molnár, Luis A. Moreno, Yannis P. Pitsiladis, Paola Russo, Alfonso Siani, Sabina Sieri, Isabelle Sioen, Toomas Veidebaum, Licia Iacoviello, on behalf of the IDEFICS consortium

**Affiliations:** 1 Research Laboratories, Fondazione di Ricerca e Cura “Giovanni Paolo II”, Università Cattolica del Sacro Cuore, Campobasso, Italy; 2 Casa di Cura Montevergine, Mercogliano (AV), Italy; 3 Department of Epidemiological Methods and Etiologic Research, Leibniz-Institute for Prevention Research and Epidemiology - BIPS GmbH, Bremen, Germany; 4 Department of Mathematics and Computer Sciences, University of Bremen, Bremen, Germany; 5 School of Life Sciences, College of Medical, Veterinary and Life Sciences, University of Glasgow, Glasgow, United Kingdom; 6 Institute for Public Health and Nursing Research, University of Bremen, Bremen, Germany; 7 Institute of Cardiovascular and Medical Sciences, College of Medical, Veterinary and Life Sciences, University of Glasgow, Glasgow, United Kingdom; 8 Research and Education Institute of Child Health, Strovolos, Cyprus; 9 Department of Pediatrics, Institute of Clinical Sciences, The Queen Silvia Children’s Hospital, Sahlgrenska Academy at University of Gothenburg, Göteborg, Sweden; 10 Department of Pediatrics, University of Pécs, Pécs, Hungary; 11 GENUD (Growth, Exercise, NUtrition and Development) Research Group, University of Zaragoza, Zaragoza, Spain; 12 Epidemiology & Population Genetics, Institute of Food Sciences, CNR, Avellino, Italy; 13 Department of Preventive and Predictive Medicine, Nutritional Epidemiology Unit, Fondazione IRCCS Istituto Nazionale dei Tumori, Milan, Italy; 14 Department of Public Health, Ghent University, Ghent, Belgium; 15 FWO, Research Foundation Flanders, Brussels, Belgium; 16 Center of Health and Behavioral Science, National Institute for Health Development, Tallinn, Estonia; 17 Department of Epidemiology and Prevention, IRCCS Istituto Neurologico Mediterraneo Neuromed, Pozzilli (IS), Italy; University of Rouen, France

## Abstract

Neuromedin U, encoded by the *NMU* gene, is a hypothalamic neuropeptide that regulates both energy metabolism and bone mass. The beta-2 adrenergic receptor, encoded by the *ADRB2* gene, mediates several effects of catecholamine hormones and neurotransmitters in bone. We investigated whether *NMU* single nucleotide polymorphisms (SNPs) and haplotypes, as well as functional *ADRB2* SNPs, are associated with bone stiffness in children from the IDEFICS cohort, also evaluating whether *NMU* and *ADRB2* interact to affect this trait. A sample of 2,274 subjects (52.5% boys, age 6.2±1.8 years) from eight European countries, having data on calcaneus bone stiffness index (SI, mean of both feet) and genotyping (*NMU* gene: rs6827359, rs12500837, rs9999653; *ADRB2* gene: rs1042713, rs1042714), was studied. After false discovery rate adjustment, SI was significantly associated with all *NMU* SNPs. rs6827359 CC homozygotes showed the strongest association (recessive model, Δ = −1.8, *p* = 0.006). Among the five retrieved haplotypes with frequencies higher than 1% (range 2.0–43.9%), the CCT haplotype (frequency = 39.7%) was associated with lower SI values (dominant model, Δ = −1.0, *p* = 0.04) as compared to the most prevalent haplotype. A non-significant decrease in SI was observed in in *ADRB2* rs1042713 GG homozygotes, while subjects carrying SI-lowering genotypes at both SNPs (frequency = 8.4%) showed much lower SI than non-carriers (Δ = −3.9, *p*<0.0001; *p* for interaction = 0.025). The association was more evident in preschool girls, in whom SI showed a curvilinear trend across ages. In subgroup analyses, rs9999653 CC *NMU* or both GG *ADRB2* genotypes were associated with either lower serum calcium or β-CrossLaps levels (*p* = 0.01). This study in European children shows, for the first time in humans, a role for *NMU* gene through interaction with *ADRB2* gene in bone strength regulation, more evident in preschool girls.

## Introduction

Bone development is a key processes characterizing growth during childhood and adolescence [1]. Understanding this process is of crucial importance for planning strategies to prevent or treat pediatric bone disorders, as well as osteoporosis later in life [2]. While it is well known that bone homeostasis is determined by the cross-talk between osteoblasts and osteoclasts, the complexity of the regulatory influences on these cells is continuously expanding [3].

Neuromedin U (NMU) is a hypothalamic neuropeptide that regulates various metabolic functions including energy homeostasis and glycemic control [4]. Recently, Sato et al. [5] showed that *NMU* double-null mice have increased bone mass, demonstrating also an interactive effect between single allele deletions of *NMU* and *beta-2-adrenergic receptor* (*ADRB2*). Thus, NMU is involved also in bone formation, acting as a central mediator of the effect of leptin, lead by sympathetic nervous system (SNS), on osteoblast ADRB2, which regulates cell proliferation [6], [7]. No candidate gene studies have been published on humans focusing on *NMU* and bone health. Moreover, the polymorphisms most significantly associated with bone health reported by genome-wide association studies (GWAS) are located in DNA regions being far from *NMU* [8]. However, GWAS mainly focus on SNPs with large effect and did not investigate all polymorphisms in all genetic models, as well as did not consider interactions among SNPs [9].

To provide more in depth knowledge on bone health in young children, this study investigated a large sample of European children of the IDEFICS study [10]. The project aimed at identifying and preventing dietary- and lifestyle-related disorders in children and infants, mainly focusing on overweight and obesity as well as on bone health disorders, also in conjunction with overweight [11], as it shares part of its risk factor profile. In the present study we investigated the association between bone stiffness and two candidate genes, *NMU* and *ADRB2*, focusing on gene-gene interactions. Moreover, we investigated whether, in subjects carrying risk alleles, a bone loss is more evident at specific ages during childhood and whether the loss interests bone mass or microarchitecture.

## Methods

### Ethics Statement

The study was conducted according to the standards of the Declaration of Helsinki. All applicable institutional and governmental regulations pertaining to the ethical use of human volunteers were followed during this research. Approval by the appropriate ethical committees was obtained by each of the eight centers engaged in the fieldwork (Belgium: Ethics Committee, University Hospital, Gent; Cyprus: Cyprus National Bioethics Committee; Estonia: Tallinn Medical Research Ethics Committee; Germany: Ethics Committee, University of Bremen; Hungary: Egészségügyi Tudományos Tanács, Pécs; Italy: Comitato Etico, ASL Avellino; Spain: Comité Ético de Investigación, Clínica de Aragón - CEICA; Sweden: Regional Ethics Review Board, University of Gothenburg). Both the children and their parents gave their oral (children) and written (parents) informed consent for examinations, collection of samples, subsequent analysis and storage of personal data and collected samples.

### Study Population

IDEFICS is a large European multi-center study on childhood obesity [10], [12]. A cohort of 16,224 children aged 2–9 years has been recruited in a population-based survey between September 2007 and May 2008 (T0), in eight European countries (Belgium, Cyprus, Estonia, Germany, Hungary, Italy, Spain and Sweden) using standardized procedures. A community-oriented intervention program for primary prevention of obesity was implemented [13]. Children were allocated to either control or intervention group and were followed up for two years (T1, 2009–2010).

DNA was extracted from a subgroup of 4,678 samples randomly selected from the total study population, stratified by country [14], [15]. All children with complete data on age, sex, parental questionnaire, height, weight, hip and waist circumferences, birthplace and language spoken at home as well as with provided saliva samples were included in the present analysis. Although no formal enquiry about ethnicity was made, two questions in the parental questionnaire provided information about ethnicity. “Place of birth of both parents” and “language habitually spoken at home” were used to select only children of European descent. DNA was successfully extracted in all cases, however, after the exclusion of samples with improbable DNA yields or not correctly genotyped, 4,641 children had at least one SNP successfully genotyped in the *NMU* (n = 4529) or *ADRB2* (n = 4566) gene. During the IDEFICS baseline survey, calcaneal quantitative ultrasound sonometry (QUS) measurements were performed in 7,447 children. The present analysis refers to the 2,267 children with genotypes and QUS data available at T0. QUS measurements were performed also two years later, during the follow-up, in 1,792 genotyped children.

### Calcaneal Bone Stiffness

Calcaneal QUS measurements were performed using Lunar Achilles Insight (GE Healthcare, Milwaukee, WI, USA) [16]. In previous studies conducted on children, coefficient of variation was 1.9–3.5% [17]–[19]. Good values of short- and long-term interunit precision were reported in a prospective multicenter study [20], [21]. Calibration of the QUS devices has been performed daily during the entire study period. Measurements were made according to the standard procedure provided by the manufacturer. The real time image of the calcaneus and the ROI parameter ensures that the measurement is accurate and alerts the examiner to perform the measure again when a child moved too much. An adapter was used for children’s feet in order to get the proper position of the calcaneus.

The device estimates calcaneal bone stiffness index (SI), calculated from broadband ultrasound attenuation (BUA) and speed of sound (SOS): SI = (0.67×BUA)+(0.28×SOS) - 420. Precision ranged from 1.0 to 3.8% (CV) for BUA and from 0.19 to 0.30% (CV) for SOS [22]. The intermediate values BUA and SOS to calculate SI were retained and registered in the database only in few centers and are available only for 878 children (T1). Both feet were measured once (100% of measures) and the mean SI of both feet was calculated and used in the statistical analyses, as well as for BUA and SOS when available.

### Anthropometric Measures

The measurement of weight was carried out using an electronic scale (Tanita BC 420 SMA, Tanita Europe GmbH, Sindelfingen, Germany) to the nearest 0.1 kg with children wearing indoor clothes, without shoes. Height was measured using a telescopic height-measuring instrument (Seca 225 stadiometer, Birmingham, UK) to the nearest 0.1 cm. The body mass index (BMI) was calculated as weight (in kg) divided by height squared (in m).

### Genotyping

Tagging SNPs of *NMU* gene were selected from the release 2.0 Phase II data of the HapMap Project (http://hapmap.ncbi.nlm.nih.gov/) using the Tagger program of Haploview software (v4.1) [23]. Selection criteria included r^2^≤0.8 and a minor allele frequency (MAF) ≥0.05 in Caucasians. *NMU* gene spans 56,156,162-56,197,222 bp (NCBI.36) on chromosome region 4q12. We selected a region that includes the third block (56,187,256-56,193,006) containing eight SNPs. Of these, three tag SNPs (rs6827359, rs12500837, rs9999653) located in intronic regions were genotyped. Two missense coding SNPs in *ADRB2* gene, rs1042713 (Arg16Gly) and rs1042714 (Gln27Glu), were selected according to previous literature on obesity risk [24], [25].

Saliva samples collected (Oragene DNA Self-Collection Kit, OG-300/OG-250; DNA Genotek Inc., Kanata, Ontario, Canada) from participating children were shipped to the central laboratory at the University of Glasgow for DNA extraction [14].

Variants of *NMU* gene were genotyped at Fondazione “Giovanni Paolo II” by a multiplexed end-point assay that detects variants of a single nucleic acid sequence. The allelic discrimination was performed by 7500 Fast Real-Time System (Applied Biosystems) using 96-well reaction plate with standard reagents and standard protocols. Result analysis was made by SDS v1.4 software (Applied Biosystems). Variants of *ADRB2* gene were genotyped at the University of Glasgow using Taqman assays (Applied Biosystems, Warrington, UK). Genotype calls were made by the analysis software (StepOne v2.1; Applied Biosystems). The genotyping success rate of the five variants examined was on average 97.6%. A random 5% repeated selection of samples for each SNP was genotyped again with 100% concordance.

### Blood Measures

Children participating in the IDEFICS survey were asked to participate, on voluntary basis, in blood drawing. Serum/plasma samples were stored at −80°C [26]. Serum calcium was determined by a standard photometric test (o-Cresolphtalein) with a Roche Cobas Integra 800 (Roche, Mannheim, Germany). Serum cross-linked collagen N-telopeptides (β-CrossLaps) and vitamin D (25(OH)D) were measured with an electrochemiluminescence immunoassay using a Roche Modular E170 analyzer (Roche). Serum leptin was determined by radioimmunoassay (Mediagnost GmbH, Reutlingen, Germany) [27].

### Statistical Analysis

The distribution of each polymorphism was assessed for deviation from Hardy-Weinberg equilibrium with chi-square test. General linear model analysis was applied to test the associations between SI and gene variants with SAS software (v9.2 for Windows, Cary, NC: SAS Institute Inc. 2002–2008) adjusting for age, sex, country and, only for T1 measurements, intervention (control *vs* intervention region). For each SNP, codominant, dominant and recessive models were tested. To select the best genetic model, we choose the genotype showing the most significant results after correction for multiple comparisons using false discovery rate (FDR) assessment. The resulting nine *p* values were imported in SAS and tested using PROC MULTTEST (INPVALUES option), which converts them in FDR adjusted values, to allow the use of standard significance cut off (p<0.05). The Haplo.stats package (v1.4.4; http://cran.r-project.org/web/packages/haplo.stats/index.html) was used to estimate the *NMU* haplotype frequencies (haplo.em function) and to verify the associations between haplotype and phenotype (haplo.glm function). The most prevalent haplotype was chosen as reference. Only the haplotypes with frequencies greater than 1% were taken into consideration for association analyses. Age, sex, country (T0) and intervention (T1 analysis) were considered as covariates. Codominant model was tested as main model, then dominant and recessive models were also tested. To investigate if an association between genotypes and SI is driven by bone mineral density rather than by trabecular structure, supplementary analyses on BUA and SOS parameters were performed in the subgroup of children with available data on these parameters.

To verify the gene-gene interaction, an interaction analysis was performed, testing the model with the two SNPs and their interaction term in the general linear model analysis. Differences in SI values were then verified between carriers and non-carriers of the most associated genotypes of both *NMU* and *ADRB2* genes.

To investigate the effect of age, sex and bone related biomarkers, further analyses were performed. The association between genotypes and SI were verified at different ages (preschool and primary school children, less than or higher than/equal to 6 years) [11] in both genders. Sex-specific SI trend during the growth was analyzed for each genotype. A locally weighted regression (LOESS) was used to test the assumption of a linear *vs.* nonlinear relationship between SI and age. SI of each child at T0 were plotted in a graph (PROC SGPLOT with LOESS statement in SAS) using a scatterplot smoothing method which automatically determines the optimal smoothing parameter [28], [29]. Adjustment for BMI or weight was performed using SI residuals obtained after regression with BMI or weight as covariates.

To give more insights in biological mechanisms, gene-environmental interactions were tested using some bone related variables as calcium (n = 605), 25-hydroxy vitamin D (n = 590), leptin (n = 252) and beta-crosslaps (n = 592) levels in serum.

## Results

### Population Characteristics

Characteristics of the study population (N = 2,267, boys 52.4%) are listed in table 1. All genotype groups were in Hardy-Weinberg equilibrium and minor allele frequencies (MAF) resulted similar to values reported in HapMap database for Caucasians (Table 2). Five haplotypes were inferred with frequencies higher than 1% (range 2.2%–43.3%; Table 3).

**Table 1:** Population characteristics.

Variables	T0			T1		
	(boys = 52.4%)			(boys = 52.3%)		
	N	Mean	SD	N	Mean	SD
Age [years]	2267	6.2	1.8	1792	8.3	1.8
Body Mass Index [kg/m^2^]	2267	16.3	2.2	1792	16.5	2.5
Weight [Kg]	2267	23.3	6.8	1792	24.0	7.2
Height [cm]	2267	118.5	13.0	1792	119.2	12.8
Stiffness index (mean of both feet)	2267	79.6	13.5	1792	82.9	13.5
Broadband ultrasound attenuation (BUA)(mean of both feet) [dB/MHz] (T1)	NA	NA	NA	865	88.2	16.7
Speed of sound (SOS ) (mean of both feet) [m/sec] (T1)	NA	NA	NA	865	1591.5	41.5
Calcium (serum) [mmol/l]	605	2.51	0.10	NA	NA	NA
25-hydroxy vitamin D (serum) [ng/ml]	590	18.26	6.80	NA	NA	NA
Leptin (serum) [ng/ml]	252	5.10	5.35	NA	NA	NA
Beta-crosslaps (serum) [ng/ml]	592	1.18	0.27	NA	NA	NA

NA = Not Available.

**Table 2:** Allele frequencies and Hardy-Weinberg Equilibrium of *NMU* and *ADRB2* gene polymorphisms (N = 2,267).

	SNP	Major:minor	Homozygousmajor allele	Heterozygous	Homozygousminor allele	*p* HWE	MAF	CEU
NMU	rs6827359	T:C	26.9%	48.8%	24.3%	0.11	0.49	0.40
NMU	rs12500837	T:C	57.3%	36.7%	6.0%	0.91	0.24	0.21
NMU	rs9999653	C:T	21.5%	49.1%	29.4%	0.42	0.54	0.49
ADRB2	rs1042713	G:A	37.0%	47.6%	15.4%	0.90	0.39	0.32
ADRB2	rs1042714	C:G	37.5%	47.2%	15.3%	0.62	0.39	0.46

*CEU*:CEPH (Utah Residents with Northern and Western European Ancestry) from International Hapmap Project.

**Table 3:** Haplotypes and haplotype frequency of the 3^rd^ *NMU* block (N = 2,267).

Haplotype			Haplotype frequency^a^
rs6827359	rs12500837	rs9999653	Total
T	T	C	43.3%
C	T	T	24.4%
C	C	T	21.9%
T	T	T	8.2%
C	C	C	2.2%

a Rare haplotypes with frequency lower than 1% were not considered (CTC, TCC and TCT, accounting for 0.05%).

### Associations between NMU and SI

Using FDR correction, four models resulted significantly associated with SI among nine tested (*p* = 0.05; Table 4) at T0. SI values were 1.5–2.5 points lower in homozygotes for the variant allele C of both rs6827359 (raw *p* = 0.006) and rs12500837 (raw *p* = 0.023), as well as in homozygotes for major allele C of rs9999653 (both dominant and codominant model, raw *p* = 0.014 and 0.020, respectively), compared to carriers of the opposite allele. The use of further covariates such as BMI or weight, plausibly related to both genotypes and SI, did not change the results. The phenotype variance explained by rs6827359 CC was 0.30%, while age plus sex explained 0.12% of variance.

**Table 4:** Differences in bone stiffness index (Δ SI) between risk and non-risk genotype carriers at T0.

Gene	SNP	Riskgenotype	Frequency	Δ SI	*P* a
*NMU*	rs6827359 T/C	CC	24.3%	−1.8	0.006^b^
*NMU*	rs12500837 T/C	CC	6.0%	−2.6	0.023^b^
*NMU*	rs9999653 C/T	CC^c^	21.5%	−1.5	0.014^b^
*NMU*	H3^d^	CCT/x	39.7%	−1.0	0.04
*NMU*	H2^d^	CTT/CTT	6.2%	−2.2	0.055
*ADRB2*	rs1042713 G/A	GG^c^	37.0%	−0.9	0.09
*ADRB2*	rs1042714 C/G	GG	15.3%	−0.6	0.37
NMU*ADRB2	rs6827359*rs1042713	CC+GG	8.4%	−3.9	<0.0001

a Adjusted for age, sex and country.

b Model selected according to the highest association after FDR correction (PROC MULTTEST in SAS software).

c Homozygotes for wild-type (instead of variant) allele were shown to concordantly retain the genotypes with lower values.

d Carriers of Haplotype TTC as reference.

The most prevalent haplotype was TTC (Table 3), then it was used as referent haplotype. Subjects carrying the CCT haplotype (containing the unfavorable allele C of both rs6827359 and rs12500837) had lower SI than those carrying the most prevalent one. This result obtained using standard additive model analysis was statistically significant (*p* = 0.04, decreased value of 1 point for each haplotype copy). Dominant and recessive models were also tested, without significant results. Homozygotes for the haplotype containing only one unfavorable allele in rs6827359 (CTT/CTT) also showed lower SI values (−2.2, *p* = 0.055, homozygosis prevalence 6.2%; Table 4).

Data on QUS measurements at T1 did not reach the statistical power needed to confirm the data, although SI values were concordantly decreased in all three unfavorable genotypes (data not shown).

### NMU-ADRB2 Interaction

Significant association between *ADRB2* genotypes and SI were not found. However, gene-gene interaction analysis considering both genotypes and their mathematical product revealed a statistically significant effect also for their interaction term (*p* = 0.025; table 4). Carriers of both the unfavorable genotypes (rs6827359 CC of *NMU* and rs1042713 GG of *ADRB2*, n = 186) were then compared with non-carriers, showing a larger difference in SI than single rs6827359 (−3.9, n = 1928, *p*<0.0001). The presence of both variants explained 0.64% of the phenotype variance. No significant effect was observed for SI values at T1.

### Sex-specific SI Trends during Growth

The interaction effect of categorical age (<6 or ≥6 years) and sex on SI was nominally significant (*p* = 0.05). Therefore, the SI differences between double opposite homozygotes in rs6827359 and rs1042713 polymorphisms were also tested in subgroups of children stratified for age and/or sex. The differences remained significant in both age classes and in both sexes. However, a further stratified analysis combining sex *per* age subgroups showed a large effect for younger girls (−7.4 points for double risk homozygotes, *p* = 0.005, p for interaction = 0.11), which was statistically significant yet after FDR adjustment (*p* = 0.021, using *p* values from the 4 subgroups of age class *per* sex), despite the decreased sample size (Table 5). The use of BMI or weight as covariates did not change the results.

**Table 5:** Bone stiffness index values in children with different combination of *NMU* rs6827359 and *ADRB2* rs1042713 alleles.

rs6827359* rs1042713	Overall			Boys						Girls					
				<6 years			≥6 years			<6 years			≥6 years		
	N	Mean	SD	N	Mean	SD	N	Mean	SD	N	Mean	SD	N	Mean	SD
**00 (TT+AA)**	1006	80.4	0.4	241	81.1	0.9	278	80.3	0.7	230	81.4	1.0	257	79.0	0.7
**01 (TT+GG)**	601	80.0	0.5	141	80.0	1.2	189	80.1	0.8	118	81.1	1.2	153	79.3	0.9
**10 (CC+AA)**	321	79.7	0.7	62	82.7	1.8	113	79.9	1.0	60	79.3	1.8	86	77.8	1.2
**11 (CC+GG)**	186	76.3	0.9	40	76.4	2.3	61	77.3	1.4	29	73.5	2.6	56	76.8	1.5
***P*** ** for interaction**	0.025			0.120			0.262			0.105			0.583		
**00+01+10**	1928	80.1	0.3	444	81.1	0.7	579	80.2	0.5	408	80.9	0.7	496	78.9	0.5
**11 (CC+GG)**	186	76.3	0.9	40	76.4	2.3	61	77. 3	1.4	29	73.5	2.6	56	76.8	1.5
**11 vs 10+01+00 Delta**	−3.9			−4.7			−2.9			−7.4			−2.1		
***P*** ** value**	0.0001			0.045			0.051			0.005			0.189		

Stiffness index values are least square means computed in a glm analysis using the variable with the four genotypes as independent variable. P for interaction was computed for CC*GG. *P* value for the association with double homozygotes for risk alleles were reported (heterozygotes were excluded).

**Figure 1:**
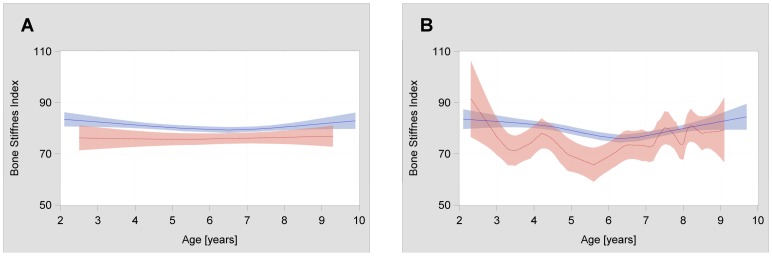
Distribution of SI values at different ages, stratified for genotypes and sex. SI values and their 95% confidence intervals at different ages for homozygotes of both *NMU* rs6827359 and *ADRB2* rs1042713 risk alleles (CC+GG, dark grey) and homozygotes for non-risk alleles (TT+AA, light gray) in subgroups of boys (panel A, n = 620, carriers of CC+GG = 101) and girls (panel B, n = 572, carriers of CC+GG = 85). Graph was obtained using SAS software (PROC SGPLOT with LOESS statement, see text). Local regression method implies that statistical power decreases at extreme *x* values (larger confidence intervals).

SI trends during age among carriers and non-carriers of double homozygosis conditions in the subgroups of girls and boys is depicted in Figure 1. Despite the similar sample size, trends in subgroups of boys (panel A) appear to be parallel, while girls (panel B) carrying both risk genotypes showed a curvilinear trend, mainly at preschool age. The trend did not become linear after adjustment for BMI or weight.

### Bone Related Parameters

BUA and SOS were available in a limited subgroup of subjects collected at T1 (n = 865). As SI, SOS values were decreased in carriers of all three unfavorable NMU genotypes, although non-significantly. Moreover, a synergistic effect of NMU and ADRB2 genotypes was observed (*p* for interaction = 0.01), with carriers of double unfavorable variants having a large decrease in SOS values (−10.7, *p* = 0.021) in comparison with non-carriers. The difference resulted larger in children younger than 6 years (−26.3, *p* = 0.008; *p* for interaction = 0.017, n = 202). No significant effect was observed for BUA values.

In a subsample ranging from 252 to 605 subjects where biomarkers were measured, homozygosity for the C allele of *NMU* rs9999653 was inversely associated with serum calcium levels, while GG genotypes of both *ADRB2* polymorphisms were associated with lower levels of β-Crosslaps (*p* = 0.01 for all). However, none of these parameters was associated with SI in this subsample (data not shown).

## Discussion

This is the first population study investigating the association between *NMU* gene and bone health and its interaction with a gene in the linked sympathetic nervous pathway involved in bone health regulation. Genetic variants of *NMU* gene are associated with SI in European children of IDEFICS population, with allele C of SNP rs6827359 showing the most significant decrease in SI. Haplotype analysis confirmed the involvement of *NMU* gene and, in particular, of SNP rs6827359, in bone health. Furthermore, there was a significant interaction between *NMU* and *ADRB2* gene, with double risk homozygous children showing a decrease of 3.9 point in SI with respect to double opposite homozygotes. The effect appeared to be more evident in younger girls, in whom a curvilinear SI trend during growth was observed. Subgroup analyses showed that the effect on SI was mainly driven by the effect on its component SOS and that the genotype-SI association was not mediated by bone related biomarkers.

NMU is a hypothalamic neuropeptide, involved in the regulation of various metabolic functions [4]. Recently, NMU was suggested to be involved also in bone health determination [5]. One of the main systems regulating bone formation involves leptin that inhibits bone formation by binding to its receptors located in hypothalamus and thereby activating the SNS. This process requires the expression of ADRB2 in osteoblasts, which mediates several effects of catecholamine hormones and neurotransmitters in bone [30]. The inhibition of bone formation through a hypothalamic relay suggested that molecules affecting energy metabolism in the hypothalamus, such as NMU, could also modulate bone mass. Using transgenic mice, Sato et al. showed that NMU acts as a modulator of leptin-SNS-ADRB2 regulation of bone formation, through a central relay and via an unidentified pathway. *NMU*-deficient mice had high bone mass due to an increase in bone formation that was reversed by a natural agonist for the NMU receptor. Furthermore, transgenic mice null for one allele of both *NMU* and *ADRB2* genes showed a more pronounced increase in bone mass, suggesting an interaction between NMU and ADRB2 [5]–[7]. We here demonstrated this phenomenon in children, showing that different SI values are associated with different *NMU* genotypes and with double homozygotes of both *NMU* and *ADRB2* risk genotypes. Thus, observations from *in vitro* data and transgenic mouse models were confirmed in humans, that NMU is involved in bone health and interacts with ADRB2 signal.

QUS is a radiation free, portable, cost-effective tool, measuring properties of bone that contribute to mechanical bone strength by sending sound waves; the more complex the bone structure, the more sound waves will be absorbed [31]. Measuring SI with QUS has value in assessing associations in children. In fact, over the last decades, many studies used QUS to study bone strength in healthy children at the calcaneus [17]–[19], [32], [33] even in preschool children [12], [34] and it was suggested to predict the risk of osteopenia or fragility fracture [33], [35]. Calcaneus is the most popular site for QUS measurement since it consists for 90% of trabecular bone and has a high turnover rate and therefore will show bone metabolic changes first [1]. Moreover, using transaxial transmission, the QUS parameters are related to bone density and structure and not to cortical thickness, which is an advantage when examining children and adolescents, since cortical thickness varies a lot during growth [36]. Finally, the safe and portable nature of the method will allow designing feasible screening programs from school to school, then calcaneus SI could became a useful marker for public health interventions.

The SI is derived from the measured values BUA, which largely reflects bone mineral density, and SOS, which reflects elasticity and density due to trabecular connectivity [37]. Unfortunately, BUA and SOS were available only at T1 and only for a subgroup of children. Although the limited sample size, SOS showed a strong association with double homozygosity of risk alleles. This result suggests that NMU and ADRB2 are involved more in microarchitecture formation than in mineral content accrual, confirming the data from Sato et al. [5], showing a prevalent role for NMU in osteoblast proliferation rather than in their function. In fact, in *NMU*-null mice they observed higher osteoblast numbers in the presence of a normal mineral apposition rate.

Despite the decreased sample size of subgroup analyses of age and sex, in girls less than 6 years significant associations were found between genotypes and SI, concordantly with the overall sample. Analyzing trends across ages, in girls a curvilinear trend was observed for carriers of both risk genotypes, while in boys SI trends appeared to be linear and parallel. Since sample sizes of boys and girls are similar, the reason of non-linearity should be mainly attributed to the major dispersion of SI values observed in pre-school girls, which suggest the involvement of other factors at this age. Anyway, the stronger association observed in younger girls could be determined by the NMU-SNS pathway and this suggests to deeply investigating the sex-specific factors potentially involved at earlier ages. These observations could be of relevance, since bone peak at early ages has been associated with future osteoporosis [2], [38]. A link between the hypothalamic neuropeptide NMU and gonadotropin secretion is known [39], which however takes places at later ages. Gender differences were also reported in NMU-deficient mice [5], where the increase in bone formation was however more prominent in male than in female mice.

Finally, we investigated possible mechanisms underlying the association between *NMU*/*ADRB2* gene and bone stiffness, by measuring biological markers related to bone metabolism in serum samples of a children subgroup. Some associations were found with calcium or β-CrossLaps levels, bone turnover markers, however they were present for SNPs less associated with SI. Furthermore, an association between SI and these biomarkers was not found, due to limited statistical power in the subgroup of genotyped subjects with biomarkers measured.

The study has some limitations.

Indeed, being the first study in humans suggesting an effect on *NMU* on bone health, it needs confirmation. The GWAS performed on bone health measured with different techniques did not reveal any effect for SNPs near *NMU* region. However, GWAS are focused on a relatively limited selection of polymorphisms which cannot be in linkage with all other known polymorphism and report only highly significant associations, as well as do not study neither all genetic model nor the interactive effects among genotypes [9]. Furthermore, heritability of QUS traits ranged from 0.42 to 0.57 [40], allowing further researches on polymorphisms with minor effects or on plausible gene-gene interactions.

The main strength of our study is a strong biological plausibility, since our results reproduce those observed in mice [5]. Moreover, it is performed on a large sample recruited in eight countries and it is focused on children, in which environmental exposure time is short and genetics should have more impact than expected.

In conclusion, our study in European children suggests an involvement of NMU-SNS pathway in bone stiffness, mainly in bone microarchitecture, more evident in preschool girls. The identification of genetic markers in the NMU pathway could be helpful in planning therapies for bone-loss disorders or metabolic diseases using novel NMU receptors inhibitors or NMU analogs [41], as well as in finding novel specific targets for preventive or therapeutic interventions.
